# Endoglin (CD105) expression differentiates between aseptic loosening and periprosthetic joint infection after total joint arthroplasty

**DOI:** 10.1186/2193-1801-3-561

**Published:** 2014-09-26

**Authors:** Philipp Jansen, Torsten Mumme, Thomas Randau, Sascha Gravius, Benita Hermanns-Sachweh

**Affiliations:** Department of Orthopedics, Medical School of the Technical University of Aachen, Aachen, Germany; Department of Orthopedic and Trauma Surgery, University of Bonn Medical School, Bonn, Germany; Institute of Pathology, Medical School of the Technical University of Aachen, Aachen, Germany; Gerhard-Schümmer-Straße 11, Geilenkirchen, 52511 Germany

**Keywords:** Endoglin, Aseptic loosening, Periprosthetic joint infection, Histopathology, Total joint arthroplasty, Periprosthetic membrane

## Abstract

The differentiation between aseptic loosening and periprosthetic joint infection (PJI) after total joint arthroplasty is essential for successful therapy. A better understanding of pathogenesis of aseptic loosening and PJI may help to prevent or treat these complications. Previous investigations revealed an increased vascularization in the periprosthetic membrane in cases of PJI via PET signals. Based on these findings our hypothesis was that PJI is associated with an increased neovascularization in the periprosthetic membrane.

Tissue samples from periprosthetic membranes of the bone-implant interface were investigated histologically for inflammation, wear particles, vascularization and fibrosis. To identify vascular structures antibodies against CD 31, CD 34, factor VIII and CD 105 (endoglin) were applied for immunohistochemical investigations. According to a consensus classification of Morawietz the tissue samples were divided into four types: type I (wear particle induced type, n = 11), type II (infectious type, n = 7), type III (combined type, n = 7) and type IV (indeterminate type, n = 7).

Patients with PJI (type II) showed a pronounced infiltration of neutrophil granulocytes in the periprosthetic membrane and an enhanced neovascularization indicated by positive immunoreaction with antibodies against CD 105 (endoglin). Tissue samples classified as type I, type III and type IV showed significantly less immune reaction for CD 105.

In cases of aseptic loosening and PJI vascularization is found in different expression in periprosthetic membranes. However, in aseptic loosening, there is nearly no neovascularization with CD 105-positive immune reaction. Therefore, endoglin (CD 105) expression allows for differentiation between aseptic loosening and PJI.

## Introduction

The demographic change in our society yielded in a more frequent need of endoprostheses. Furthermore, total joint arthroplasties due to arthrosis become more and more common at younger ages as there is a progressive incidence of obesity in society. In Germany, around 150,000 total hip arthroplasties are implanted each year and approximately 1.3 million worldwide (Berry et al. 2000; Katzer and Löhr 2003). 10 years after implantation endoprostheses survivorship rates of 88-95% in total hip replacements and 93% in total knee arthroplasties could be proven (Argenson et al. 2013; Allami et al. 2006). After 15 years implant survival rates of only 58-62% in total hip arthroplasties and 79% in total knee replacements were described (Mäkelä et al. 2011; Newman et al. 2009). The most common reasons for failure are aseptic loosening and periprosthetic joint infection (PJI) (Lima et al. 2013; Jiang et al. 2013). The differentiation between both complications remains a key challenge in orthopedic surgery as the treatment of aseptic loosening is completely different to the treatment of PJI (Ellenrieder et al. 2011).

So far, there is no single accepted diagnostic tool to differentiate between aseptic loosening and PJI. Various diagnostic tests have been proposed and current recommendations are based on several pre-, intra-, and postoperative tests (Miyamae et al. 2013; Sendi and Zimmerli 2012).

Systemic inflammation markers such as erythrocyte sedimentation rate (ESR), C-reactive protein (CRP) serum level and white blood-cell count play a substantial role in the differentiation between aseptic loosening and PJI, but they are not consistently reliable as they are highly sensitive but less specific (Parvizi et al. 2011; Costa et al. 2012). Cytological investigations of joint aspirations are suggestive for a PJI with a leukocyte count greater than 1,700 to 3,000 leukocytes per ml and with an amount of more than 60% neutrophils in the synovial fluid (Mason et al. 2013; Dinneen et al. 2013). Results of microbiologic examinations of joint aspirations can yield false-negative results in 12% (Parvizi et al. 2008).

Microbiological cultures and histopathological examination of the periprosthetic membrane of the bone-implant-interface are crucial procedures to differentiate between aseptic loosening and PJI (Larsen et al. 2012). Intraoperative cultures show a broad range of sensitivity (12-100%) and specifity (81-100%) which is caused by different incubation times, choice of media as well as sample contamination (Krukemeyer 2009).

Histopathological examinations of the periprosthetic membrane proved a valid diagnostic tool with a high sensitivity and specifity to detect a PJI. A consensus classification published by Morawietz et al. ( 2006) allows the differentiation of four types of loosening membranes (*type I* (wear particle-induced type), *type II* (infectious type), *type III* (combined type) and *type IV* (indeterminate type)) with a high accuracy (0.94) in identification of PJI.

In conclusion, none of these tests is 100% reliable. False-positive or negative results could lead to a misdiagnosis with crucial consequences for the treatment. Consequently, there is a need for further research and development into new methods aimed at improving diagnostic accuracy.

In this context, previous investigations revealed increased vascularization in periprosthetic membranes via PET signals (Mumme et al. 2003a, 2005, 2003b, Reinartz et al. 2005; Kisielinski et al. 2003). Concurrent, there is an intensive expression of endoglin (CD105) during angiogenesis, especially angioneogenesis in wound healing and inflammation (Torsney et al. 2002). Endoglin is a homodimer transmembrane protein. Its expression is regulated by hypoxia and by binding of TGF-β to endothelium cells offering receptors for TGF-β1 and β3. In contrast to other vascular markers (factor VIII, etc) endoglin only stains immature, newly formed vessels (Cheifetz et al. 1992).

The examination of angiogenesis in the periprosthetic membrane could be a helpful tool to differentiate between aseptic loosening and PJI. So far, a study that examines the expression of endoglin in periprosthetic membranes has not been described. This study should create more understanding of the pathogenesis of aseptic loosening and PJI by investigating angiogenesis histologically and immunohistochemically in accordance to the consensus classification of Morawietz. Our hypothesis was that PJI is associated with an increased neovascularization in the periprosthetic membrane and endoglin is a helpful biomarker to differentiate between aseptic loosening and PJI.

## Materials and methods

### Patient population

In this study, 32 consecutive patients were included who underwent revision surgery due to aseptic loosening or PJI after total hip or knee arthroplasty: 29 with total hip arthroplasties and 3 with total knee arthroplasties. Used materials were polyethylene (PE) and ceramic. 17 patients were male with an average age of 65.4 (48-78) years and 15 female with an average age of 69.5 (49-84) years. In 6 cases one or more revision surgeries have been done before. The mean duration from primary arthroplasty to revision surgery was 12.0 years in type I (4-23 years), 4.3 years in type II (1-12 years), 10.8 years in type III (2-15 years) and 8.6 years in type IV (1-21 years). Preoperatively the laboratory inflammation marker CRP was determined. In 16 out of 32 cases FDG-PET investigations were done beforehand. Indications for PET investigations were suspicion of septic or aseptic loosening of total hip arthroplasties and a time period of at least 12 months postoperatively. PET investigations were not done in cases of highly septic patients with urgent surgery indication. Out of those 16 cases type I was represented with 7/11, type II with 4/7, type III with 4/7 and type IV with 1/7. In 14 cases microbiological examinations were performed.

### Pathological examination

All samples were taken from periprosthetic membranes from the bone-implant-interface during revision surgery in cases of aseptic loosening or PJI. Formalin-fixed and paraffin-embedded material was stained by hematoxylin and eosin for routine procedures. Inflammation, foreign-body reaction, neutrophil granulocytes, lymphocytes, macrophages, foreign-body giant cells, fibrosis, vascularization and wear debris were evaluated semi-quantitatively in a score system from 0-3 (0 = not existing, 1 = slightly distinctive, 2 = moderately distinctive, 3 = highly distinctive) in the entire specimen (400× magnification). Tissue samples were classified into the different types due to histological criteria.

### Immunohistochemical investigations

Immunohistochemical investigations were performed on paraffin sections. Antigen retrieval was done with citrate buffer at ph 6.0 in a cooking pot. Endogenous peroxidase was blocked with hydrogen peroxidase. For visualization the EnVision™ system (Dako) was applied which uses a streptavidin-biotin polymer conjugated secondary antibody with DAB (diaminobenzidine), both per Dako’s instructions. Antibodies against CD 31 (dilution: 1:500; DAKO, Hamburg, Germany) and CD 34 (dilution: 1:200; DAKO, Hamburg, Germany) which stain mature as well as immature vessels were used to illustrate the whole of all vessels. Factor VIII (dilution: 1:300; DAKO, Hamburg, Germany) was applied to mark mature vessel endothelium and show pre-existing vessels. In contrast endoglin (CD 105) (dilution: 1:200; DAKO, Hamburg, Germany) only stains immature which means newly formed vessels. Positive and negative controls were performed. According to Dako’s instructions positive controls of endoglin (CD 105) were done with tissue samples of lymph nodes. The entire immunohistochemical specimens were examined in 400× magnification with the same semi-quantitative score system used for evaluation in hematoxylin and eosin staining.

### Statistical analysis

For statistical analysis the program SAS (Version 9.2, Institute Inc., Cary, NC, USA) was used. Median, minimum, maximum, mean value, Kruskal-Wallis test, Wilcoxon test, Spearman’s correlation coefficient and weighted kappa were performed. The Kruskal-Wallis test was used to compare group 1, 2, 3 and 4. In case of a significant p calculated with the Kruskal-Wallis test pair comparisons were performed with the help of the Wilcoxon test. All tests were performed two-sided. The evaluation was done exploratory: this means all p values <0.05 were considered as significant.

## Results

### Clinical information

1. Relationship of CRP and consensus classification
  The laboratory inflammation marker CRP is in normal range in the majority of patients with type I (8/11) and type III loosening (4/7) (elevated specimens: type I: 12-143 mg/l; type III: 30-130 mg/l). CRP elevations can be proven in 7/7 patients with type II loosening (30-164 mg/l) and 5/7 with type IV loosening (6-217 mg/l). There are no false-negative results concerning elevation of CRP and TEP loosening of the infectious type. Including type III loosening to the group of PJI 4/7 CRP values are false-negative. In the group of aseptic loosening (type I and type IV) there were 8 false-positive results. Overall CRP shows a sensitivity of 71% and a specifity of 56%. Concerning type I and type IV loosening sensitivities of 73% and 29% are proven. Regarding type II and type III loosening specifities of 73% and 43% are verified.
2. Microbiological analyses
  7 out of 14 microbiological examinations were positive (1/2 in type I, 3/4 in type II, 2/5 in type III, 1/3 in type IV). False-positive results in type I and type IV might be due to contamination. False-negative test results can be caused by inadequate incubation time, inappropriate choice of media and antimicrobial therapy.
3. FDG-PET
  3/4 PET investigations in cases of type II loosening showed signs of inflammation (sensitivity 75%). 7/7 PET investigations in type I, 4/4 in type III and 1/1 in type IV were negative concerning signs of inflammation. A previous FDG-PET investigation proved a sensitivity of 94%, a specifity of 95% and an accuracy of 95% in discriminating between infection and loosening (Reinartz et al. 2005).

### Light microscopy

Tissue samples of the periprosthetic membrane of the bone-implant-interface of all patients were investigated lightmicroscopically and immunohistochemically. The specimens show periarticular tissue with synovia and subsynovial structures, fat as well as connective tissue with vessels and nerval fascicles. Some show fibrosis. In diverging degree inflammation cells can be found. Depending on the level of tissue reaction, inflammation and the amount of wear debris they are categorized using a categorization developed by Morawietz et al. ( 2006). Furthermore, wear particles were classified by a particle algorhythm published by Krenn et al. ( 2014).

**Figure 1:**
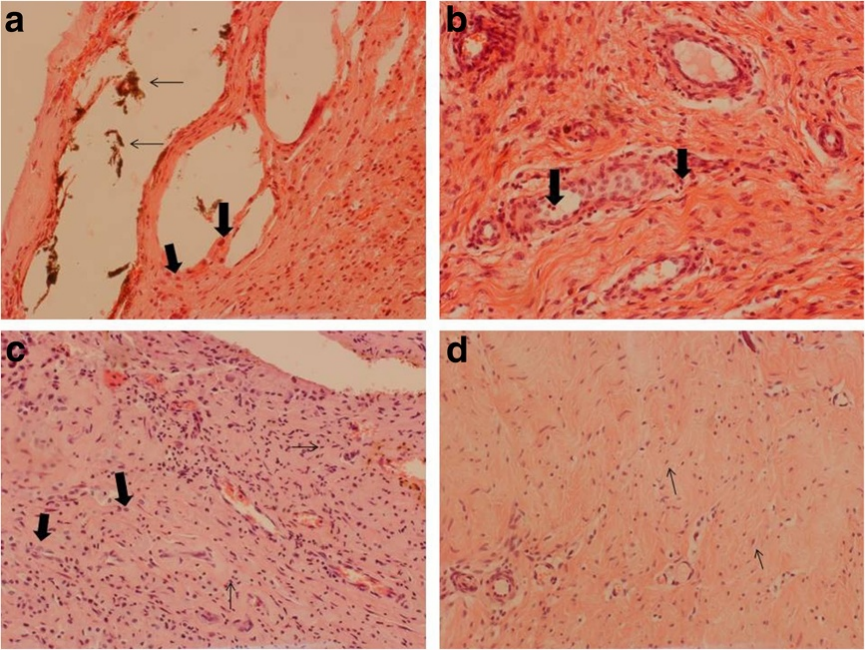
**a-d Overview of all types of periprosthetic membranes in H & E staining.** Magnification: 200x. **a** Type I (wear particle-induced type) with wear debris (marked with thin arrow) (macro-particular PMMA in hollows of PE particles, no non-implant material, no necrosis, no lymphocytary infiltration) and surrounding foreign-body reaction with macrophages (marked with thick arrow) and foreign-body giant cells. **b** Type II (infectious type) with high density of vessels and granulocyte infiltration (marked with arrow). **c** Type III (combined type) showing characteristics of the wear particle-induced type and infectious type, less macrophages (thick arrow) and granulocytes (thin arrow). **d** Type IV (indeterminate type) with connective tissue, fibrocytes and fibroblasts (marked with arrow); no inflammatory cells.

The periprosthetic membrane of the *wear particle-induced type (type I, n = 11)* as shown in Figure 1a is characterized by macrophages and foreign-body giant cells. Some show fibrosis. There are small wear particles in macrophages and big particles incorporated in multinuclear giant cells. Wear debris is displayed birefringent in polarized light. Lymphocytes are rarely found.

There are no granulocytes in the periprosthetic membrane after semiquantitative evaluation of the examined tissue samples (mean value 0.0, standard deviation 0.0). Lymphocytes are expressed with a score of 0.73 (standard deviation 0.47), macrophages with a score of 2.91 (standard deviation 0.30), foreign-body giant cells with a score of 2.64 (standard deviation 0.50), vascularization with a score of 1.0 (standard deviation 0.0), fibrosis with a score of 2.09 (standard deviation 1.30) and wear debris (mainly macroparticles of polyethylene (PE) and bone cement (PMMA), no non-implant material, few necrosis, scattered lymphocytes) with a score of 2.18 (standard deviation 0.75).

The periprosthetic membrane of the *infectious type (type II, n = 7)* which is indicated in Figure 1b is characterized by a granulation tissue consisting of capillary proliferation, fibroblasts and neutrophil granulocytes.

Granulocytes are evaluated with a score of 2.43 (standard deviation 0.53), lymphocytes with a score of 1.43 (standard deviation 0.79), macrophages with a score of 2.43 (standard deviation 0.53), foreign-body giant cells with a score of 1.29 (standard deviation 0.95), vascularization with a score of 2.71 (standard deviation 0.49), fibrosis with a score of 2.43 (standard deviation 0.79) and wear debris (mainly microparticles of PE, bone fragments, no necrosis, 1/7 with extensive lymphocyte infiltration) with score of 1.57 (standard deviation 1.27).

The periprosthetic membrane of the *combined type (type III, n = 7)* is demonstrated in Figure 1c. It consists in equal parts of granulocyte dominated granulation tissue of the infectious type and of macrophage and foreign-body giant cell dominated wear particle-induced type.

Granulocytes are expressed with a score of 1.29 (standard deviation 0.49), lymphocytes with a sore of 1.71 (standard deviation 0.95), macrophages with a score of 2.71 (standard deviation 0.49), foreign-body giant cells with a score of 1.57 (standard deviation 0.79), vascularization with a score of 1.29 (standard deviation 0.49), fibrosis with a score of 1.43 (standard deviation 0.98) and wear debris (mainly macroparticles of PE and PMMA, bone fragments, few necrosis, 1/7 with extensive lymphocyte infiltration) with a score of 2.0 (standard deviation 0.82).

The periprosthetic membrane of the *indeterminate type (type IV, n = 7)* as displayed in Figure 1d is characterized by a membrane that consists of cell-poor connective tissues bordered by a thin margin of fibrin with fibroblasts and macrophages. A few neutrophil granulocytes are localized in this margin of fibrin. Wear particles are hardly found.

Granulocytes are evaluated with a score of 0.71 (standard deviation 0.49), lymphocytes with a score of 2.86 (standard deviation 0.38), macrophages with a score of 1.86 (standard deviation 0.38), foreign-body giant cells with a score of 0.29 (standard deviation 0.49), vascularization with a score of 1.71 (standard deviation 0.49), fibrosis with a score of 2.29 (standard deviation 0.49) and wear debris (mainly microparticles of PE, bone fragments, no necrosis, extensive lymphocyte infiltration) with a score of 1.14 (standard deviation 0.69).

### Immunohistochemistry

**Figure 2:**
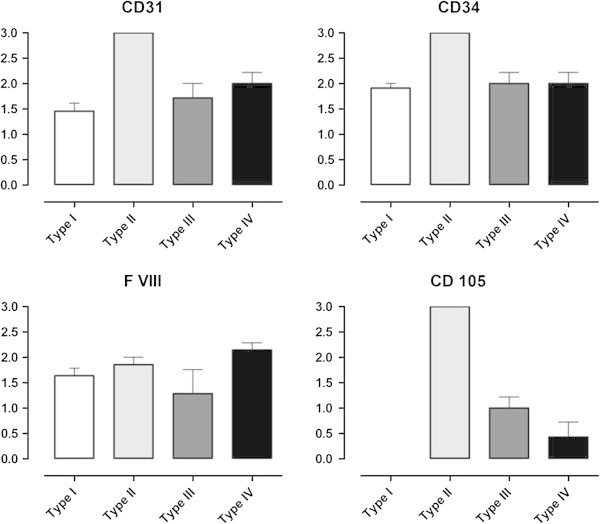
**Immunohistochemical analysis concerning vascularization in periprosthetic membranes (type I-IV).** Degree of expression: 0-3. Vascular markers: CD 31, CD 34, CD 105 (endoglin), factor VIII. CD 105 (endoglin) demonstrates newly formed vessels and is expressed significantly more in cases of PJI (type II) (p < 0.0001).

Subsequent to the findings in light microscopy we performed immunohistochemical investigations to compare the amount of newly formed vessels, pre-existing vessels and vessels in general. Both CD 31 and CD 34 were used as pan-endothelium cell makers. They visualize a highly distinctive vascularization in type II (7/7 tissue samples express CD 31 and CD 34 highly distinctive (mean value 3.0, standard deviation 0.0)), a slightly to moderately distinctive vascularization in type I (mean values: CD 31: 1.45, CD 34: 1.91, standard deviations: CD 31: 0.52, CD 34: 0.30) and a moderately distinctive vascularization in type III and IV (mean values: CD 31 in type III: 1.71, CD 34 in type III: 2.00; CD 31 in type IV: 2.00, CD 34 in type IV: 2.00, standard deviations: CD 31 in type III: 0.76, CD 34 in type III: 0.58; CD 31 in type IV: 0.58, CD 34 in type IV: 0.58). These results are displayed in Figure 2.

Factor VIII pointes out a moderate distinctive level of pre-existing vessels in all types (mean values: type I: 1.64, type II: 1.86, type III: 1.29, type IV: 2.14, standard deviations: type I: 0.50, type II: 0.38, type III: 1.25, type IV: 0.38) which is shown in Figure 2.

**Figure 3:**
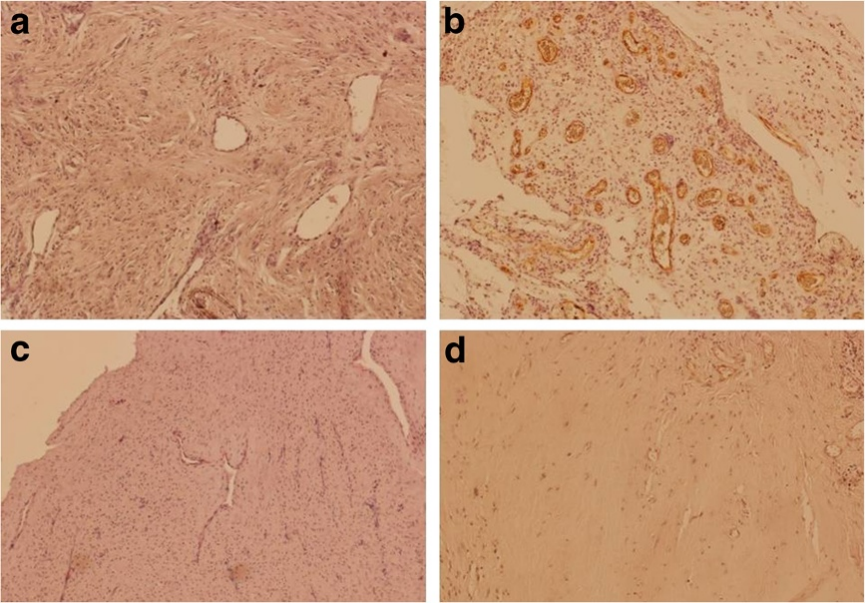
**Immunohistochemical staining with CD 105 of all types of periprosthetic membranes.** Magnification: 100x. **a** Type I (wear particle induced type) without proof of CD 105-positive endothelium. **b** Type II (infectious type) with high amount of CD 105-positive endothelium. **c** Type III (combined type) with slightly distinctive proof of CD 105. **d** Type IV (indeterminate type) without proof of CD 105.

**Figure 4:**
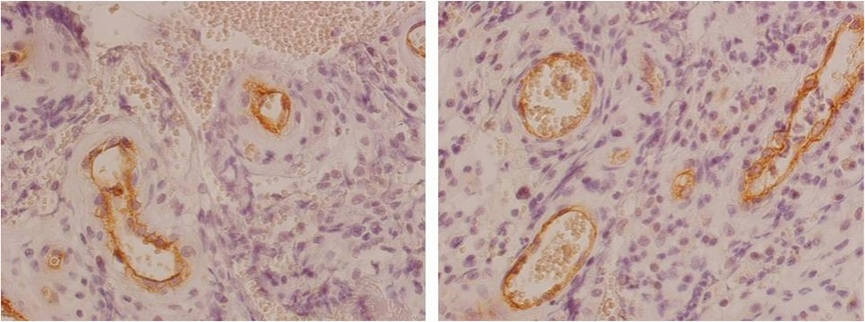
**Immunohistochemical staining with CD 105 in two high power fields of type II (infectious type) loosening.** Magnification: 400x. CD 105 stains vessel endothelium which is surrounded by a highly density of inflammatory cell infiltration.

Figure 3a-d illustrates an immunohistochemical cytoplastmatic staining of endothelial cells of newly formed vessels with CD 105 in periprosthetic membranes. Those newly formed vessels can be proven in areas of active inflammation – primarily with an extensive density of granulocytes (see Figure 4). CD 105-positive vascularization is highly distinctive in type II (mean value 3.0, standard deviation 0.0) and slightly distinctive in type III (mean value 1.0, standard deviation 0.58). CD 105 is not expressed in type I (mean value 0.0, standard deviation 0.0) and slightly in type IV membranes (mean value 0.43, standard deviation 0.79).

### Statistical analysis

The degree of CD 31, CD 34 and CD 105 is expressed more in type II than in the other types. After statistical analysis the immunohistochemical impression can be underlined statistically (CD 31: p = 0.0006, CD 34: p = 0.0003, CD 105 p < 0.0001). CD 105 is also expressed significantly more in type III in comparison to type I and IV (p < 0.0001).

Spearman’s coefficient of correlation (CC) emphasizes that expression of granulocytes and CD 105 are accompanied by each other (CC = 0.83).

### Essentials

Light microscopy proves the existence of inflammatory cells, fibrosis and vascular proliferation in different expression in all types of periprosthetic membranes. But the highest degree of inflammation and newly formed vessels can be found in the infectious type (type II). Fibrosis is found moderately expressed in the wear particle-induced type (type I), infectious type (type II) and indeterminate type (type IV) and slightly expressed in the combined type (type III). Histological and immunohistochemical investigations demonstrate a floride inflammation process accompanied by neovascularization is predominantly found in PJI (type II) with a significant expression of endoglin (CD 105).

## Discussion

The correct identification of a periprosthetic joint infection after total hip or knee arthroplasty is often difficult as the clinical symptoms often resemble those of aseptic loosening. A false-positive diagnosis of PJI could lead to an unnecessary two-stage exchange (girdlestone-situation) or a cemented revision (one-stage exchange). On the other hand a misdiagnosis of PJI would result in a one-stage revision without appropriate treatment, most likely resulting in persisting infection and early implant failure (Parvizi et al 2010). In cases of clinical signs of PJI treatment should be initiated within the first 48 hours. Otherwise probabilities of treatment failure rise significantly (Brandt et al. 1997; Hsieh et al. 2009). There are a lot of studies attempting to identify the best combination of diagnostic procedures predicting PJI. The current literature has highlighted the need of further research in this field (Miyamae et al 2013; Sendi and Zimmerli 2012).

The hypothesis of our study was that there is an increased vascularization in periprosthetic membranes during PJI in contrast to aseptic loosening of total hip or knee arthroplasty. As previous investigations proved increased vascularization in periprosthetic membranes via PET signals (Mumme et al. 2003a, 2005, 2003b; Reinartz et al. 2005; Kisielinski et al. 2003), this study should create more understanding of both complications by investigating angiogenesis histologically and immunohistochemically. Guo et al. ( 2006) describe a positive correlation between PET-signals and detection of endoglin-positive newly formed vessels in lung adenocarcinomas. Furthermore, it is well known that there is an increased expression of endoglin during angiogenesis, especially angioneogenesis in wound healing and inflammation (Torsney et al. 2002). A study that examines the expression of endoglin in periprosthetic membranes of loosened prostheses has not been described yet.

This study investigated tissue samples of the periprosthetic membrane of the bone-implant-interface of 32 patients. Signs of chronic inflammation are found in all types of membranes. A foreign-body reaction is predominantly found in type I membranes (wear particle-induced type), whereas a floride inflammation process led by neutrophils is found in type II membranes (infectious type). Fibrosis can be detected in all types of membranes and is proven in most specimens. These results were represented in Morawietz’ consensus classification ( 2006).

Vascularization of small and large vessels proven by CD 31 and CD 34 are found in all types. But the highest density of vessels is proven in type II (infectious type). Increased vascularization in PJI was also described by Pessler et al. ( 2008). To characterize vascularization more detailed we investigated the vascular profile with the help of antibodies against pre-existing vessels (factor VIII) and newly formed vessels (endoglin (CD 105)). It could be verified that type II (infectious type) membranes do not show more pre-existing vessels but significantly more newly formed vessels. These newly formed vessels can be proven by endoglin (CD 105). Periprosthetic membranes of type III loosening represent a chronic PJI in comparison to the extended inflammatory infiltrate in our collective of type II membranes. Granulocytes and endoglin are expressed significantly less in comparison to type II loosening, but significantly more in comparison to type I and type IV loosening. Thus, endoglin allows differentiating between aseptic loosening and PJI. Our study underlines the results of studies by Mumme et al. who proved increased cell infiltration and vascularization in PJI by FDG-PET (Mumme et al. 2003a, 2005, 2003b; Reinartz et al. 2005). The role of endoglin in inflammatory processes was also described by Rossi et al. ( 2013). Immunohistochemical detection of endoglin-positive angiogenesis was demonstrated in specimens of patients with lung cancer by Guo et al. ( 2006) and Takase et al. ( 2010).

In addition, a correlation between neutrophil granulocytes and endoglin (CD 105) can be shown. Pessler et al. ( 2008) described that infiltration of granulocytes and neovascularization correlate with each other in chronic septic arthritis as well as in septic total arthroplasties. Neutrophil granulocytes produce cytokines in the process of neovascularization (Vågesjö et al. 2012). In our study a correlation between granulocytes and neovascularization could be proven in acute phases of infection (type II membranes) as well as in chronic infections (type III membranes).

Diagnostic synovial biopsies in periprosthetic loosening are a clinically applied diagnostic tool. Its high diagnostic value was described by Fink et al. ( 2013). We could demonstrate that immunohistochemical investigations of the periprosthetic membrane with endoglin can support the differentiation between aseptic loosening and PJI. Patients with PJI (periprosthetic membrane of the infectious type) show a pronounced infiltration of neutrophil granulocytes in the periprosthetic membrane and an enhanced neovascularization indicated by positive immunoreaction with antibodies against CD 105 (endoglin). By investigating neovascularization histologically and immunohistochemically we could demonstrate first that newly formed vessels detected by endoglin (CD 105) are predominantly found in PJI and that endoglin expression allows differentiating between aseptic loosening and PJI.

As a consequence of the presented results it would be interesting to establish endoglin as a diagnostic tool for preoperative differentiation between aseptic loosening and PJI because tissue samples of the periprosthetic membrane out of the bone-implant-interface are not accessible without exchange of the prosthesis.

The value of immunohistochemical endoglin detection has already been demonstrated in the field of tumor diagnostics (Dallas et al. 2008). From tumor research we know that the serum level of endoglin is significantly elevated in people with metastatic cancer and primary tumors such as hepatocellular carcinomas (Takahashi et al. 2001; Clasper et al. 2013; Elnemr et al. 2012). It is focus of current investigations whether a serum level determination of endoglin could be used in diagnostics of prosthesis loosening.
